# De Novo Design of Peptidic Positive Allosteric Modulators Targeting TRPV1 with Analgesic Effects

**DOI:** 10.1002/advs.202101716

**Published:** 2021-07-11

**Authors:** Lizhen Xu, Heng Zhang, Yunfei Wang, Xiancui Lu, Zhenye Zhao, Cheng Ma, Shilong Yang, Vladimir Yarov‐Yarovoy, Yuhua Tian, Jie Zheng, Fan Yang

**Affiliations:** ^1^ Kidney Disease Center First Affiliated Hospital and Department of Biophysics Zhejiang University School of Medicine Hangzhou Zhejiang 310058 China; ^2^ Alibaba‐Zhejiang University Joint Research Center of Future Digital Healthcare Hangzhou Zhejiang 310058 China; ^3^ MOE Frontier Science Center for Brain Science and Brain‐Machine Integration Zhejiang University Hangzhou Zhejiang 310027 China; ^4^ College of Wildlife and Protected Area Northeast Forestry University Harbin 150040 China; ^5^ Protein facility School of Medicine Zhejiang University Hangzhou Zhejiang 310027 China; ^6^ Department of Physiology and Membrane Biology University of California Davis School of Medicine Davis CA 95616 USA; ^7^ Qingdao University School of Pharmacy Qingdao Shandong 266101 China

**Keywords:** ion channel, pain, positive allosteric modulator, protein design, TRPV1

## Abstract

Transient receptor potential vanilloid 1 (TRPV1) ion channel is a nociceptor critically involved in pain sensation. Direct blockade of TRPV1 exhibits significant analgesic effects but also incurs severe side effects such as hyperthermia, causing failures of TRPV1 inhibitors in clinical trials. In order to selectively target TRPV1 channels that are actively involved in pain‐sensing, peptidic positive allosteric modulators (PAMs) based on the high‐resolution structure of the TRPV1 intracellular ankyrin‐repeat like domain are de novo designed. The hotspot centric approach is optimized for protein design; its usage in Rosetta increases the success rate in protein binder design. It is demonstrated experimentally, with a combination of fluorescence resonance energy transfer (FRET) imaging, surface plasmon resonance, and patch‐clamp recording, that the designed PAMs bind to TRPV1 with nanomolar affinity and allosterically enhance its response to ligand activation as it is designed. It is further demonstrated that the designed PAM exhibits long‐lasting in vivo analgesic effects in rats without changing their body temperature, suggesting that they have potentials for developing into novel analgesics.

## Graphical abstract

Transient receptor potential vanilloid 1 (TRPV1) ion channel is critically involved in pain sensation. Positive allosteric modulators (PAMs) of TRPV1 are de novo designed based on the structure of TRPV1. Surface plasmon resonance and patch‐clamp recording demonstrate that the PAMs bind to TRPV1 and allosterically enhance its activities. The PAM exhibits analgesic effects without changing body temperature in rats.

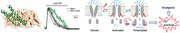

## 1 Introduction

Chronic pain is more than an unpleasant feeling, it can be so devastating that not only the quality of life in patients is drastically lowered but also enormous social‐economical costs are imposed. For instance, in the United States alone, over 100 million adults suffer from chronic pain with an annual economic cost of nearly 600 billion dollars.^[^ ^1^ ^]^ Though analgesics such as opioids and nonsteroidal anti‐inflammatory drugs are available, their low efficacy against chronic pain, side effects, and the complex nature of pain demand developments of novel analgesic drugs.

The transient receptor potential vanilloid 1 (TRPV1) ion channel is a prototypical sensor involved in nociception,^[^ ^2^ ^]^ making it a promising target for pain managements.^[^ ^3^ ^]^ Indeed, genetically knocking out this channel leads to much reduction in thermal hyperalgesia.^[^ ^4^ ^]^ Antagonizing TRPV1 pharmacologically also effectively alleviates dental, rectal, and thermal pain.^[^ ^5^, ^6^ ^]^ However, because TRPV1 channel is a polymodal receptor activated by heat and involved in body temperature regulation, systematic blockade of this channel incurred substantial hyperthermia in clinical trials, thus impeding further drug developments.^[^ ^5^, ^6^, ^7^ ^]^ TRPV1 agonists such as resiniferatoxin potently ablate TRPV1‐expressing neurons in the dorsal root and trigeminal ganglia by inducing calcium overload in these neurons,^[^ ^8^ ^]^ but such an analgesic approach is irreversible so that its application is currently limited to intractable cancer pain.^[^ ^9^ ^]^ Therefore, to develop analgesics against chronic pain with both reversibility and reduced adverse effects such as hyperthermia, novel strategies to modulate TRPV1 activities are needed.

Instead of using agonists or antagonists to modulate TRPV1 activity universally, positive allosteric modulators (PAMs) that selectively modulate the high‐activity population of TRPV1 are a promising alternative. TRPV1 channel is a calcium permeable channel highly expressed in nociceptive nerve termini.^[^ ^2^ ^]^ Previous studies have established that positive allosteric modulation of TRPV1 activities leads to local calcium overload in nociceptive afferent nerve terminus, causing functional and reversible inactivation of the nerve terminus to exert analgesic effects. For instance, the small molecule PAM of TRPV1 MRS1477, which was discovered in structure‐activity relationship studies,^[^ ^10^ ^]^ enhances TRPV1 activation in the presence of orthosteric agonists such as capsaicin and exerts analgesic effects.^[^ ^11^, ^12^ ^]^

To develop effective PAMs for TRPV1, we used peptidic design approach instead of performing resource‐consuming screening campaigns. Our method took advantage of the rich information from structural and functional investigations in TRPV1^[^ ^13^, ^14^ ^]^ and the rapid evolving computational protein design approach.^[^ ^15^, ^16^ ^]^ Specifically we chose the hotspot centric Rosetta computational approach which enables rational design of protein binders to a specific domain of any target of a known 3D structure.^[^ ^17^ ^]^ Using this approach, previous studies reported successful de novo design of proteins bound to the stem region of hemaglutinin and the Fc domain of IgG.^[^ ^16^, ^17^, ^18^ ^]^ For TRPV1, previous electrophysiological studies have shown that its ankyrin repeat‐like domain (ARD) is critically involved in ligand induced desensitization.^[^ ^13^, ^14^, ^15^, ^16^, ^17^, ^18^, ^19^, ^20^ ^]^ We first improved the optimized hotspot centric approach (OHCA) protein design strategy to increase the success rate of obtaining robust designed binders. We then applied the improved Rosetta protein design approach to precisely target the ARD of TRPV1 to achieve positive allosteric modulation. Moreover, both the crystal structure of the ARD and the cryo‐electron microscopy structures of TRPV1 have been determined.^[^ ^13^, ^14^, ^15^, ^16^, ^17^, ^18^, ^19^, ^20^, ^21^ ^]^ With a combination of our OHCA design, fluorescence resonance energy transfer (FRET) imaging, protein chemistry, surface plasmon resonance (SPR), patch‐clamp recordings, and animal behavioral tests, we demonstrated that two out of three of our designed PAMs bind to the ARD of TRPV1 with about 70 nanomolar affinity to positively modulate this channel in cells. Furthermore, we demonstrated that in rats one of our PAMs exerts longer lasting analgesic effects as compared to MRS1477 without inducing hyperthermia.

## 2 Results

### 2.1 De Novo Design of PAMs with OHCA

**Figure 1:**
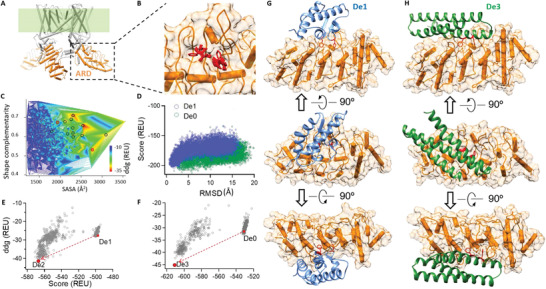
Computational design of protein binders to the ARD of TRPV1. A) Sideview of the structure of TRPV1 channel shown as cylinders (PDB ID: 3J5P). The transmembrane domains are shaded in green. The intracellular ARD is highlighted in orange. B) Two phenylalanine residues (colored in red) docked to the concave surface of ARD serve as the hot spots to anchor the binder to the ARD. C) Multimetric evaluation of initial binder designs (dots colored in gray) with shape complementarity, SASA, and ddg. D) Refolding of the candidate designs with ab initio modeling. The designed structures serve as the reference to calculate and plot the root mean square deviation (RMSD) of models versus their total score in Rosetta energy unit (REU). E,F) In silico affinity maturation of the candidate designs. Both the total score and ddg are optimized. G,H) Final designs (*De1* and *De3* in blue and green, respectively) bound with the ARD (surface in orange).

To positively modulate TRPV1 channel in a precise and domain‐specific manner, we chose to design binders to the ARD (**Figure** **1A**). The major steps in our OHCA protein design approach were illustrated in a flow chart (Figure S1, Supporting Information), where we employed three steps in computation (colored in blue) to eliminate pseudo positive candidates and increase the success rate of binder design. Similar to the original hotspot centric method,^[^ ^16^ ^]^ to first anchor hotspot residues on the ARD for further design, we docked each natural amino acid to the concave surface of ARD (Figure 1B). Among these residues, we observed that two phenylalanine residues bound to ARD with favorable binding energy and structural convergence in the sidechain conformation (Figure 1B), so we employed these two phenylalanine residues as the hotspots. We then selected a pool of protein structures as candidate scaffolds based on multiple criteria (see the Experimental Section for details). With Patchdock software^[^ ^22^ ^]^ and the Rosetta suite,^[^ ^15^ ^]^ we fused the candidate scaffolds with the two hotspots based on protein shape complementarity, and then redesigned the residues on candidate scaffolds forming the interface with TRPV1 ARD for larger binding energy (the score term ddg in Rosetta suite). At this step, thousands of initial candidate binder designs were generated (Figure 1C, dots in gray).

To increase the success rate of our design trials in the downstream experimental validations, we need to eliminate pseudo positive designs while keep the promising designs among the initial candidates. Toward this goal, we optimized the original hotspot centric method in three consecutive steps. First, we evaluated the candidate designs multimetrically (Figure 1C). The candidate designs were sorted based on their shape complementarity, binding energy (ddg), and solvent accessible surface area (SASA) of the interface (Figure 1C). Only candidates with shape complementarity, ddg, and SASA larger than 0.6, −25 Rosetta Energy Unit (REU) and 1000 Å^2^, respectively, were kept. Among these candidates, designs with top 50 ddg were further selected. We inspected these candidates and found 14 unique scaffolds. In the second step, we evaluated whether these 14 candidates could refold toward the designed structures. Because when the scaffolds were fused with the hotspots and their interface residues were redesigned, the protein sequence homology of a candidate was reduced to about 70% of its scaffold, raising the risk of large changes of structural stability. We performed the classic ab initio modeling of these candidates based on their designed primary sequence only. We observed that only two (*De0*, which is the precursor of *De3;* and *De1*) out of the 14 candidates could refold back to the designed 3D structures with a funnel‐shaped energy distribution (Figure 1D), whereas the other 12 designs exhibited either a positive score which indicated no proper refolding, or nonfunnel‐shaped score distribution in refolding (Figure S2, Supporting Information). Based on these observations, we focused on *De0* and *De1* in the subsequent work. In the third step, we performed in silico affinity maturation^[^ ^23^ ^]^ to simultaneously optimize the binding energy (ddg) and the stability (total score) of these two designs. For instance, *De2* was the optimized version of *De1*, whose ddg and stability scores were both better than those of *De1*. *De0* was optimized to yield *De3* (Figure 1E,F, see the Experimental Section for details). In *De1* and *De3*, the hotspot residues (F36 and F83 of *De1*, F72 and F76 of *De3*) were designed to interact with the concave surface of the ARD through hydrophobic interactions (Figure 1G,H). Finally, we chose three designs (*De1*, De2, and *De3*) for experimental validation (Table S1, Supporting Information), using another design (*De4*, which could not refold well) as a negative control (Figure S2, Supporting Information).

### 2.2 Designed Proteins Bind with the ARD of TRPV1

**Figure 2:**
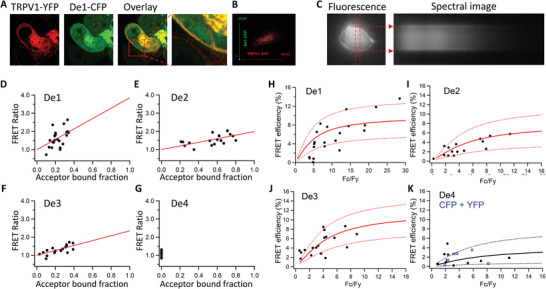
Interaction of the designed proteins with TRPV1 channel. A) Colocalization of TRPV1‐YFP and De1‐CFP in HEK293 cells revealed by Airyscan super resolution imaging. TRPV1‐YFP and De1‐CFP are pseudo‐colored in red and green, respectively. B) The 2D histogram of fluorescence signal from TRPV1‐CFP and De1‐YFP exhibited a strong colocalization. C) FRET imaging of TRPV1‐YFP and De1‐CFP coexpressed in HEK293 cells. The emission spectra measured from the edge of cell (dotted arrows in red) are used for FRET efficiency calculation. D–G) The filled circles indicate the measured FRET Ratio values for cells coexpressing the fluorophore‐tagged TRPV1 and designed proteins. The red line indicates the predicted FRET Ratio values. H–K) The FRET efficiency measured from cells coexpressing the fluorophore‐tagged TRPV1 and designed proteins. The efficiency value was plotted as a function of the fluorescence intensity ratio between CFP and YFP. Each symbol represents a single cell. The solid curve represents the FRET model that yields the best fit; dotted curves represent models with 5% higher or lower FRET efficiencies. In (K), FRET efficiency values from coexpression of CFP and YFP (open box in blue) were overlaid.

To experimentally test whether our designed proteins work as PAM for TRPV1, we first tested whether they could interact with TRPV1 channel in live cells. We genetically fused yellow fluorescent protein (YFP) and cyan fluorescent protein (CFP) to the C termini of TRPV1 and our designs, respectively. When *De1* and TRPV1 channel were cotransfected and expressed in HEK293 cells, we observed clear colocalization of these molecules on cell membrane in Airyscan super resolution imaging (**Figure** **2A**). Indeed, the 2D histogram of fluorescence signal^[^ ^24^ ^]^ from TRPV1‐CFP and De1‐YFP exhibited a strong colocalization of 95.2% ± 5.4% of the colocalization area (*n* = 11) (Figure 2B). Due to the limitation in spatial resolution of the Airyscan imaging (about 120 nm laterally),^[^ ^25^ ^]^ we further performed FRET imaging because FRET occurs only when the distance between donor and acceptor fluorophores is less than 10 nm.^[^ ^26^ ^]^ By imaging the emission spectra of design‐CFP and TRPV1‐YFP coexpressed in cells measured from the plasma membrane region (Figure 2C), we detected positive FRET signals from all three designs (*De1*, *De2*, and *De3*) and no FRET between *De4*, the negative control, and TRPV1 (Figure 2D–G and Table S2, Supporting Information). We further quantified the FRET efficiency between the designed protein and TRPV1 using the spectraFRET method we previously used to study TRP channels.^[^ ^27^ ^]^ We observed that *De1, De2*, and *De3* show FRET efficiency values of 9.2%, 7.4%, and 10.9%, respectively (Figure 2H–J), much larger than that of De4 (3.8%, Figure 2K), which was indistinguishable from the negative control where standalone CFP and YFP proteins were coexpressed (Figure 2J, points in gray for *De4* and in blue for the CFP + YFP negative control). Therefore, our imaging experiments showed that the designs interact with TRPV1 channel in live cells.

**Figure 3:**
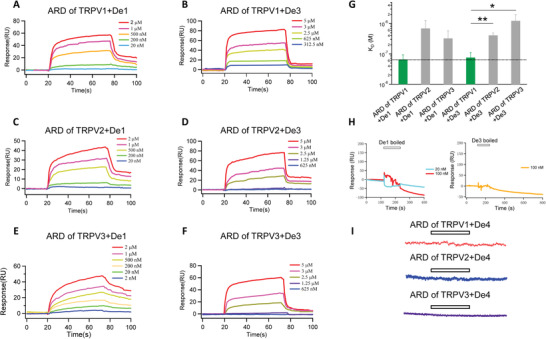
Binding of the designed proteins to the ARD of TRPV channels. A–F) SPR signal traces of *De1* and *De3* at distinct concentration levels bound to the ARD of TRPV1, TRPV2, or TRPV3 fixed on the sensor chip, respectively. G) Measurements of *K*_D_ values from the kinetics of SPR signals (*n* = 4–8) *: *p* < 0.05; **: *p* < 0.01 in *t*‐test. All statistical data are given as mean ± s.e.m. H) No SPR signal was detected when *De1* and *De3* are first boiled to disrupt their 3D structures, respectively. I) No SPR signal was detected between *De4* and the ARD of TRPV channels.

To further quantify the binding affinity of the designs to the ARD, we expressed and purified both designed proteins and the ARD of TRPV1, TRPV2, and TRPV3 in *Escherichia coli* (*E. coli*). The proteins of ARDs, *De1*, *De3*, and *De4* were robustly and abundantly produced in *E. coli*. (Figure S3, Supporting Information), while the expression of *De2* was minimal in bacterial cells. We then quantified the binding of *De1* and *De3* to ARD proteins fixed on a sensor chip by SPR (**Figure** **3A**,B). We observed that *De1* and *De3* bound with the ARD of TRPV1 in a concentration‐dependent manner, yielding *K*_D_ values of 65.5 × 10^−9^ ± 28.1 × 10^−9^ and 76.0 × 10^−9^ ± 35.2 × 10^−9^ m, respectively (Figure 3C,D). As the ARDs in TRPV1, TRPV2, and TRPV3 are highly conserved (Figure S4, Supporting Information), it is not surprising that *De1* and *De3* also bound to these ARDs (Figure 3C–F). However, the *K*_D_ values of *De1* and *De3* binding to TRPV1 ARD were much smaller than those to ARDs of TRPV2 or TRPV3 (Figure 3G, bars in green and gray, respectively), indicating that our designs showed higher selectivity for TRPV1. As negative controls, when *De1* and *De3* proteins were boiled to disrupt their 3D structures, the binding with the ARD was completely abolished (Figure 3H). Moreover, in agreement with the FRET experiments, our negative control design *De4* did not show any SPR binding signal in ARD of TRPV1, TRPV2, or TRPV3 (Figure 3I). These results demonstrated that *De1* and *De3* can potently and selectively bind with the target ARD as we designed.

### 2.3 Designed Proteins are PAMs of TRPV1

**Figure 4:**
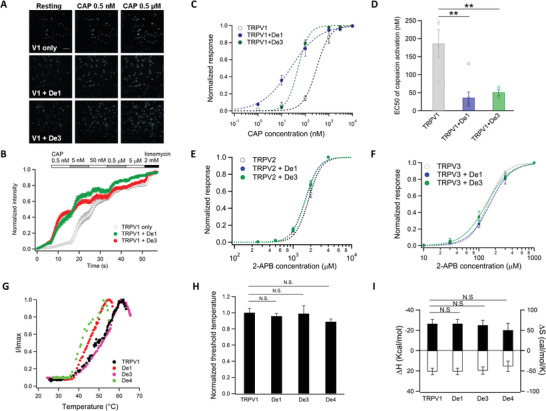
*De1* and *De3* selectively potentiate ligand activation of TRPV1. A) Representative calcium imaging of capsaicin activation of TRPV1 alone or coexpressed with the designed proteins. Scale bar: 40 µm. Cells are pseudo colored. B) Concentration dependence of capsaicin activation of TRPV1 alone or coexpressed with the designed proteins in calcium imaging experiments (*n* = 14–19). All statistical data are given as mean ± s.e.m. C) Concentration response curves and D) EC50 values of capsaicin activation of TRPV1 alone or coexpressed with the designed proteins in whole‐cell patch clamp recordings (*n* = 4–7). **: *p* < 0.01 in *t*‐test. Concentration response curves of 2‐APB activation of E) TRPV2 and F) TRPV3 alone or coexpressed with the designed proteins in whole‐cell patch clamp recordings (*n* = 4–7). All statistical data are given as mean ± s.e.m. G) Representative heat activation of TRPV1 and TRPV1 coexpressed with *De1, De3*, or *De4*. H) Heat activation threshold temperature of TRPV1 coexpressed with designed proteins was normalized to that of TRPV1 channel expressed alone (*n* = 3–7). N.S. indicates no significance. I) Changes in enthalpy (Δ*H*) and entropy (Δ*S*) of TRPV1 and TRPV1 coexpressed with *De1, De3*, or *De4* (*n* = 3–7). N.S. indicates no significance. All statistical data are given as mean ± s.e.m.

We investigated whether TRPV1 activation could be positively modulated by the designed proteins. With calcium imaging, we observed that, while 0.5 × 10^−9^ m capsaicin did not elicit detectable calcium influx in cells expressing only TRPV1, this low concentration was sufficient to activate TRPV1 channels coexpressed with either *De1* or *De3* (**Figure** **4A**,B), indicating the designed proteins enhanced capsaicin sensitivity in TRPV1. Consistent with calcium imaging results, patch‐clamp recordings revealed that, when either *De1* or *De3* was coexpressed with TRPV1, the concentration‐response curve of capsaicin activation in the absence of extracellular calcium was left‐shifted (Figure 4C), with the EC50 values reduced from 186.1 × 10^−9^ ± 38.8 × 10^−9^ to 50.9 × 10^−9^ ± 7.8 × 10^−9^ or 36.1 × 10^−9^ ± 16.1 × 10^−9^ m, respectively (Figure 4D). In contrast, when the designed proteins were coexpressed with the closely related ARD‐containing TRPV2 or TRPV3 channels, no change in ligand activation was observed (Figure 4E,F), indicating our designed proteins were highly selective for the TRPV1 channel. These results indicated our designs can positively and selectively modulate TRPV1 activities.

**Figure 5:**
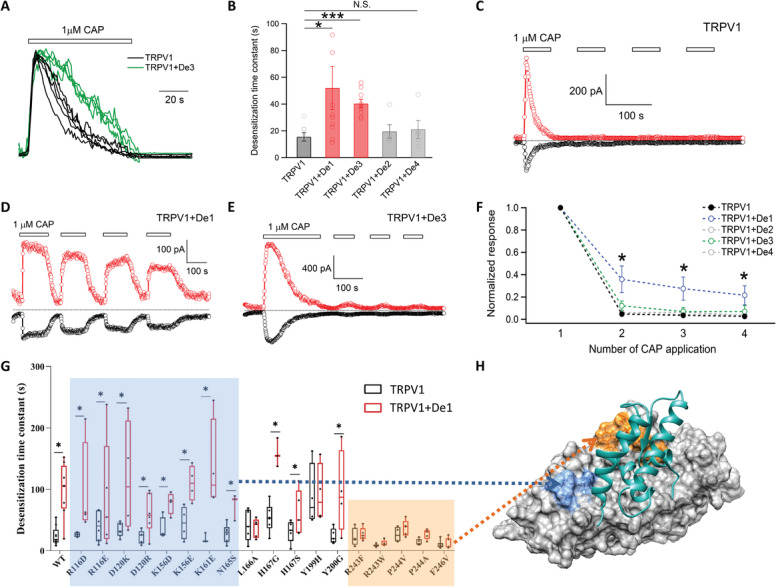
*De1* and *De3* alleviate acute desensitization and tachyphylaxis in ligand activation of TRPV1. A) Current traces of capsaicin activation and acute desensitization of TRPV1 alone (black) or coexpressed with *De3* (green) in the presence of extracellular calcium (1 × 10^−3^ m). B) The time constants of acute desensitization of TRPV1 alone or coexpressed with the designed proteins (*n* = 4–9). * and ***: *p* < 0.05 and *p* < 0.001 in *t*‐test, respectively. N.S., no significance in *t*‐tests. Representative current recordings of C) capsaicin induced tachyphylaxis of TRPV1 alone or D,E) coexpressed with the designed proteins. F) Measurements of tachyphylaxis as the current response amplitudes of the repeated capsaicin applications normalized to the first application. *: *p* < 0.05 in *t*‐test. All statistical data are given as mean ± s.e.m. G) Box and whisker plot of the time constants of acute desensitization of TRPV1 and mutants alone or coexpressed with De1 (box in black and red, respectively) (*n* = 3–7). *: *p* < 0.05 in *t*‐test. All statistical data are given as mean ± s.e.m. The mutants that maintained and abolished the increase of desensitization time constant by *De1* were shaded in blue and orange, respectively. H) The mutants that maintained and abolished the increase of desensitization time constant by *De1* were mapped to the ARD of TRPV1. The same color scheme was used as in (G).

Moreover, ligand activation of TRPV1 was accompanied with an acute desensitization process in the presence of extracellular calcium ions (**Figure** **5A**, current traces in black), which serves as a negative feedback mechanism to prevent excessive calcium entry through the channel causing calcium overload and cell damages. The designed PAM proteins were expected to exert analgesic effects by countering such a desensitization during ligand activation. We observed that when either *De1* or *De3* was coexpressed with TRPV1, current desensitization upon capsaicin activation was significantly slowed down (Figure 5A,B), whereas no such an effect was observed when *De2* or *De4* was coexpressed. Besides the acute desensitization in TRPV1 ligand activation, repeated agonist application also led to tachyphylaxis in TRPV1 channel, where the current responses exhibited diminishing amplitudes^[^ ^13^ ^]^ (Figure 5C). We observed that in the presence of *De1*, capsaicin induced tachyphylaxis was reduced (Figure 5D,F), which again increased calcium entry through the channel. *De3* did not reduce tachyphylaxis of TRPV1 (Figure 5E,F). Therefore, both calcium imaging and patch‐clamp recording demonstrated that *De1* and *De3* are PAMs of TRPV1. Furthermore, the slowed acute desensitization and reduced tachyphylaxis with our designed PAMs indicated more calcium entry through TRPV1 channels, which formed the basis for local calcium overload and analgesia.

As these PAMs were designed to bind to the ARD of TRPV1, we employed alanine scan mutagenesis in the ARD to systematically investigate whether they bind as we designed. While coexpression of *De1* with wildtype TRPV1 largely increased the desensitization time constant, we observed that mutations to TRPV1 residues at the interface between *De1* and ARD (Figure 5H, molecules in green and gray, respectively), such as R243, P244, and F246 (Figure 5G,H, residues shaded in orange), largely abolished the increase in desensitization time constant in the presence of *De1*. In contrast, mutations to residues outside the binding interface, such as R116, D120, K156, and H167 (Figure 5G,H, residues shaded in blue), did not affect the slowing down of TRPV1 desensitization by *De1*. Therefore, the results of mutagenesis experiments support the binding configuration of our designed PAM.

### 2.4 Designed PAMs Exert Analgesic Effects

**Figure 6:**
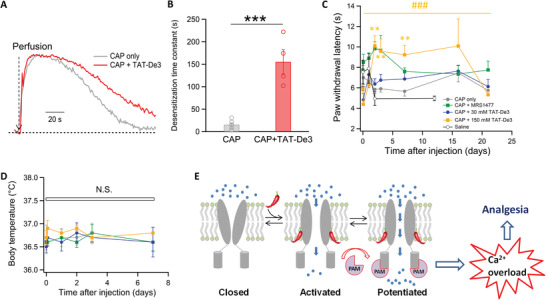
*TAT‐De3* exerts analgesic effects against heat pain in rats. A) Representative whole‐cell current recordings of capsaicin activation and acute desensitization of TRPV1 alone (gray) or with *TAT‐De3* (1 × 10^−6^ m) perfused extracellularly (red) in the presence of extracellular calcium (1 × 10^−3^ m). B) The time constants of acute desensitization of TRPV1 alone or with *TAT‐De3* perfused extracellularly. *: *p* < 0.05 in *t*‐test. All statistical data are given as mean ± s.e.m. C) Changes in paw withdrawal latency in rats injected with different ligands in heat pain tests. Paw withdrawal latency is normalized to the value measured before injection (*n* = 9–13). *: *p* < 0.05 in *t*‐test. ^#^: *p* < 0.05 in one‐way analysis of variance (ANOVA) tests. All statistical data are given as mean ± s.e.m. D) Body temperature of rats measured in the course of heat pain tests. N.S., no significance in two‐way ANOVA tests (*n* = 3–6). All statistical data are given as mean ± s.e.m. E) A cartoon diagram illustrating the mechanisms underlying positive allosteric modulation of TRPV1 and analgesia.

To test whether our designed PAMs have analgesic effects, we performed animal behavior experiments. Since the ARD of TRPV1 is located intracellularly, we genetically fused the transactivator of transcription (TAT) peptide (RKKRRQRRR)^[^ ^28^ ^]^ to the N terminus of *De1* and *De3* to facilitate their transmembrane delivery. Only *TAT‐De3* was able to be expressed and purified in *E. coli*. We observed that the *TAT‐De3* can still bind to the ARD in SPR experiments, though its affinity was reduced (Figure S5A, Supporting Information). Up to 300 × 10^−6^ m *TAT‐De3* did not disrupt the cell membrane even after 30 min incubation (Figure S5B, Supporting Information). More importantly, *TAT‐De3* applied extracellularly could diffuse across the cell membrane to slow down capsaicin induced acute current desensitization (**Figure** **6A**,B) just like when *De3* was applied intracellularly (Figure 5A,B).

We observed significant analgesic effects against thermal pain when *TAT‐De3* was intradermally injected into the hind paw of rats. As a positive control, we used the small molecule PAM MRS1477, whose analgesic effects against thermal pain has been demonstrated.^[^ ^11^ ^]^ We focused the lightbeam from a halogen lamp to a spot less than 20 mm^2^ on the rat hind paw to raise local temperature above 45 °C to elicit thermal pain. We recorded paw withdrawal latency to reflect the pain sensation. Injection of saline did not alter the latency (Figure 6C, open circles in black). Injection of capsaicin alone (50 µL at 150 × 10^−6^ m) caused initial acute pain, but after two hours when we started to measure heat induced paw lifting behavior, we observed no change in the paw withdrawal latency (Figure 6C, solid circles in gray). After coinjection of MRS1477 and capsaicin (50 × 10^−6^ and 150 × 10^−6^ m, respectively; total volume of 100 µl), we observed the paw withdrawal latency was increased after two hours; such an analgesic effect diminished after 3 d (Figure 6C, solid squares in green). After injection of *TAT‐De3* and capsaicin (150 × 10^−6^ and 150 × 10^−6^ m, respectively; total volume of 100 µl), we found that our designed proteins exerted analgesic effects to the similar magnitude as MRS1477 (Figure 6C, solid squares in orange). Though the onset of such analgesic effects was later than that of MRS1477, after a single injection they lasted for as long as 16 d, which was much longer than that of MRS1477. Importantly, injection of PAMs (either MRS1477 or *TAT‐De3*) did not cause hyperthermia in rats (Figure 6D).

## 3 Discussion

We have successfully de novo designed PAMs of TRPV1 targeting its ARD with a modified OHCA method; these PAMs exerted analgesic effects against thermal pain in rats. ARD is a classic protein domain mediating protein‐protein and protein‐ligand interactions.^[^ ^29^ ^]^ In particular, the ARD of TRPV1 is known to bind with small molecules such as adenosine triphosphate and interact with the C terminus to regulate channel desensitization,^[^ ^13^, ^14^, ^15^, ^16^, ^17^, ^18^, ^19^, ^20^ ^]^ making this domain attractive for developments of allosteric modulators for TRPV1. High resolution structures of both the ARD and TRPV1 channel have been determined, which laid a solid foundation for our structure‐guided de novo design efforts.^[^ ^13^, ^14^ ^]^ With all these knowledge, we employed a Rosetta computational protein design approach to make modulators of TRPV1 in a precise and domain‐specific manner. The ARD in TRP channels are highly conserved (Figure S4, Supporting Information), we reasoned that the specific positive allosteric regulation of TRPV1 channel by our designed proteins is not only due to the preferential binding to TRPV1 ARD (Figure 3) but also the high specificity of capsaicin to TRPV1, as both our designed proteins and capsaicin are needed to exert analgesic effects. As structures of more and more proteins are resolved with the rapid advancing structural biology techniques, we believe that de novo design of domain‐specific binders using the structure‐guided computational protein design approach will continue to gain its momentum for developing novel therapeutics.

To further improve computational design methods and increase success rate in designing protein binders, we developed an improved OHCA method. In the original hotspot centric design study, only two out of 87 (2.2%) designed candidates exhibited positive binding to hemagglutinin in experimental tests.^[^ ^16^ ^]^ As protein design approaches evolved, when protein binders to IgG were designed, nine out of 17 candidates exhibited positive binding in experimental validation,^[^ ^18^ ^]^ improving the success rate to about 50%. For our OHCA approach, with more stringent filtering of the designs by three computed metrics (shape complementarity, ddg and SASA), ab initio refolding and in silico affinity maturation of the designs, three in four designs exhibited positive binding to TRPV1 channel in cells (Figure 2). As the scoring functions and sampling methods keep evolving, we believe both the success rate and the quality of designs will be further improved.

TRPV1 is a well‐known target for analgesic drug developments. Pharmacological inhibition of TRPV1 channel effectively alleviate several types of pain in clinical trials, but systematic administration of TRPV1 inhibitors led to side effects such as hyperthermia.^[^ ^5^, ^6^, ^7^ ^]^ Such setbacks in developing TRPV1‐targeting analgesics suggest that instead of direct antagonism of this channel, inactivation and ablation of TRPV1‐expressing, pain‐sensing nerve termini by enhancing TRPV1 activities became a rational alternative. For instance, recently a synthetic capsaicin analog CNTX‐4975 has been developed to activate TRPV1 for the purpose of inactivating local pain‐sensing nerve fibers.^[^ ^30^ ^]^ Local administration of CNTX‐4975 has been effective against pain in the knee associated with osteoarthritis in a Phase 2b clinical trial.^[^ ^30^ ^]^ Moreover, PAMs of TRPV1 such as MRS1477 and our *TAT‐De3* have also demonstrated analgesics effects in animal behavioral studies.^[^ ^11^ ^]^ Though protein drugs often requires injection for drug delivery, and immunological responses to injected proteins may be triggered during repeated application, with the advance in peptide packaging and delivery, such risks will be reduced. Therefore, we believe that synergizing with the advancements in structure biology to determine architectures of drug targets and computational techniques to design binders, our de novo designed, domain‐specific binders such as *De3* will serve as a valid starting point for novel analgesic drug development in the future.

## 4 Experimental Section

### Molecular Biology and Cell transfection

Murine TRPV1, TRPV2, and TRPV3 (gifts from Dr. Michael X. Zhu at University of Texas Health Science Center at Houston, Dr. Itaru Kojima at Gunma University and Dr. Ardem Patapoutian at Scripps Research Institute, respectively) were used in this study. The cDNA of these channels was inserted into the pEYFP‐N3 vector, so that eYFP was fused to the C terminus of TRPV channels to help identify channel‐expressing cells. Tagging of eYFP did not change the functional properties of TRPV1 as it was reported before.^[^ ^27^ ^]^ Point mutations were generated by QuickChange II mutagenesis kit (Agilent Technologies). All mutants were confirmed by DNA sequencing.

HEK293T cells were purchased from American type culture collection (ATCC). These cells were authenticated to be contamination‐free by ATCC. Cells were cultured in a Dulbecco's modified eagle medium with 10% fetal bovine serum and 20 × 10^−3^ m l‐glutamine at 37 °C with 5% CO_2_. cDNA constructs of channels were transiently transfected with Lipofectamine 2000 (Life technologies) according to the manufacturer's protocol. One or 2 d after transfection, electrophysiological recordings were performed.

### Chemicals

All chemicals were purchased from Sigma‐Aldrich unless otherwise stated.

### Computational Design of Protein Binders to the ARD

Protein binders to the ARD were de novo designed following the hotspot centric stratagem described previously (Figure S1, Supporting Information).^[^ ^16^ ^]^ Briefly, the structure of TRPV1 ARD (PDB ID: 2PNN) was first cleaned and relaxed in the Rosetta suite version 3.4^[^ ^15^ ^]^ (For technical details, see Script S1, Supporting Information). Then each of the natural amino acids was docked to ARD in Rosetta.^[^ ^31^ ^]^ Two Phe residues bound well to the ARD so that they were chosen as the hotspots for subsequent protein design. The inverse rotamer library of these two residues was generated using RosettaScripts^[^ ^23^ ^]^ (Script S2, Supporting Information). The scaffold library was generated by selecting protein structures from the protein data bank (PDB) database with the following criteria:

1) There is not any DNA, RNA or disulfide bond.
2) There is only one protein chain and stoichiometry is monomer.
3) There are less than 100 residues.
4) It can be expressed in *E. coli*.
5) The structure is determined by X‐ray at a resolution higher than 2.5 Å.
6) There is no ligand presented in structure.
7) Homologue removal is set at 70% identity.

A total of 167 unique protein structures was selected. They were further cleaned and prepacked in Rosetta (Scripts S3 and S4, Supporting Information). The ARD was then docked to the scaffold library in a coarse‐grained manner with the PatchDock software^[^ ^22^ ^]^ based on protein structure shape complementarity. The patchdocked scaffolds were fused with the two hotspots and then the protein‐protein interface was redesigned by RosettaScripts (Script S5, Supporting Information). These initial designs were again screened. Only the designs with shape complementarity, ddg, and SASA larger than 0.6, −25 REU and 1000 Å^2^, respectively, were kept. 14 designs with unique protein scaffolds were selected. These designs were further ab initio refolded based on their primary sequence in Rosetta. Only two designs exhibited funnel‐shaped energy‐root mean square deviation (RMSD) distribution, indicating reasonable refolding. There two designs were then subjected to in silico affinity maturation by RosettaScripts (Script S6, Supporting Information). The designs with the largest *ddg* and *score* were selected as the final designs.

All the molecular graphics of protein structure models were rendered by University of California San Francisco Chimera^[^ ^32^ ^]^ software version 1.12.

### Fluorescence Imaging and FRET Quantification

Super‐resolution fluorescence microscopy was performed with the Zeiss LSM 880 fluorescence microscope with Airyscan. YFP fused to TRPV1 and CFP fused to the designed proteins were excited by laser line at 488 and 458 nm, respectively. A 60x oil‐immersion objective (numerical aperture 1.42) was used in these experiments. Fluorescence imaging was performed with the default settings for CFP and YFP channels in the microscope controlled by the ZEISS efficient navigation software. Overlay of YFP‐ and CFP‐channel images was also done in ZEN.

For spectroscopic imaging in FRET experiments, the imaging system was built upon a Nikon TE2000‐U microscope. The excitation light was generated by an Ar laser. The duration of light exposure was controlled by a computer‐driven mechanical shutter (Uniblitz). A spectrograph (Acton SpectraPro 2150i) was used in conjunction with a charge coupled device (CCD) camera (Roper Cascade 128B). In this recording mode two filter cubes (Chroma) were used to collect spectroscopic images from each cell (excitation, dichroic): cube I, D436/20, 455dclp; cube II, HQ500/20, Q515lp. No emission filter was used in these cubes. Under the experimental conditions, auto fluorescence from untransfected cells was negligible. Fluorescence imaging and analysis were done using the MetaMorph software (Universal Imaging). User‐designed macros were used for automatic collection of the bright field cell image, the fluorescence cell image, and the spectroscopic image. Emission spectra were collected from the plasma membrane of the cell by positioning the spectrograph slit across a cell and recording the fluorescence intensity at the position corresponding to the membrane region (Figure 2C, dotted lines in red); the same slit position applied to both the spectrum taken with the CFP excitation and the spectrum taken with the YFP excitation. Using this approach, the spectral and positional information are well preserved, thus allowing reliable quantification of FRET efficiency specifically from the cell membrane. Spectra were corrected for background light, which was estimated from the blank region of the same image.

FRET data was quantified in two ways. Frist, the FRET ratio was calculated from the increase in YFP emission due to energy transfer as described in the previous study.^[^ ^33^ ^]^ Briefly, CFP emission was separated from YFP emission by fitting of standard spectra acquired from cells expressing only YFP or CFP. The fraction of YFP‐tagged molecules that are associated with CFP‐tagged molecules, Ab, is calculated as Ab = 1/(1 + *K*_D_/[Dfree]), where *K*_D_ is the dissociation constant and [Dfree] is the concentration of free donor molecules. Note that FRET Ratio = 1 + Ab * (FRET Ratio_max_ − 1). Regression analysis was used to estimate Ab in individual cells. From each cell, the FRET ratio_exp_ was experimentally determined. The predicted Ab value was then computed by adjusting two parameters, FRET Ratio_max_ and apparent *K*_D._ Ab was in turn used to give a predicted FRET ratio_predicted_. By minimizing the squared errors (FRET ratio_exp –_ FRET ratio_predicted_)^2^, *K*_D_ was determined.

Second, apparent FRET efficiency was also calculated from the enhancement of YFP fluorescence emission due to energy transfer^[^ ^27^, ^28^, ^29^, ^30^, ^31^, ^32^, ^33^, ^34^ ^]^ using a method as it was previously described.^[^ ^35^ ^]^ Briefly, RatioA_0_ and RatioA_1_ were measured to calculate FRET efficiency. RatioA_0_ represents the ratio between tetramethylrhodamine maleimide emission intensities (in the absence of fluorescein maleimide) upon excitation at the donor and acceptor excitation wavelengths,^[^ ^34^, ^35^, ^36^ ^]^ and was calculated in the present study at the YFP peak emission wavelength. A particular advantage of quantifying RatioA_0_ for FRET measurement is that changes in fluorescence intensity caused by many experimental factors can be cancelled out by the ratiometric measurement. A similar ratio, termed RatioA, was determined in the presence of CFP in the same way as RatioA_0_. If FRET occurred, the RatioA value should be higher than RatioA_0_; the difference between RatioA and RatioA_0_ was directly proportional to the FRET efficiency by the factor of extinction coefficient ratio of CFP and YFP.^[^ ^34^, ^35^, ^36^ ^]^

### Protein Expression and SPR Measurements

The DNA sequence of each designed proteins and the ARD of mouse TRPV1, TRPV2, and TRPV3 (see their primary protein sequences in Table S3, Supporting Information) was synthesized and inserted into the pET‐32a plasmid after codon optimization for protein expression in bacteria*. E. coli* BL21 (DE3) was transformed with the recombinant plasmids. A single colony was inoculated into media containing ampicillin; cultures were incubated in 37 °C at 200 rpm. Once cell density reached to optical density 0.8–1.0 at 600 nm, isopropyl β‐D‐thiogalactoside (IPTG) was introduced for induction. Sodium dodecyl sulfate – polyacrylamide gel electrophoresis was used to monitor the expression. A range of expression conditions are tested, where the best protein expression condition was determined to be induction with 0.5 × 10^−3^ m IPTG and expression at 15 °C for four hours in lysogeny broth medium. The protein expression was stopped by centrifugation at 8000 rpm for 30 min. Cells were collected and resuspended in resuspension solution containing 50 × 10^−3^ m Tris‐HCl, 150 × 10^−3^ m NaCl, 10% glycerol and protease inhibitor (pH 7.4). The cells were lysated by sonication. The supernatant after centrifugation was kept for future purifications. Taget proteins were obtained by Ni column, and then further purified by the reversed phase high performance liquid chromatography on a Jupiter C4 column (10 × 250 mm, Phenomenex, Torrance, CA, USA). The system was equilibrated by 0.1% trifluoroacetic acid (TFA) (Solution A). The proteins were separated with gradient of 0.1% TFA acetonitrile (Solution B) at a rate of 1.5 mL min^−1^.

The purified ARD was immobilized on the CM5 SPR sensor chip by an amine‐coupling procedure. SPR experiments were conducted on the Biacore T3000 instrument (GE healthcare). The designed proteins were perfused as analytes. The kinetics in SPR responses were measured and used to determine the *K*_D_ values. In all experiments, the analysis was performed at 25 °C, with an association time of 120 s at a flow rate of 20 µL min^−1^.

Brevibacillus choshinensis is a protein‐hyperproducing bacterium. Thioredoxin (TrxA) genes were inserted from Brevibacillus choshinensis before designed proteins and the gene was expressed in *Escherichia coli* with a hexa‐His‐tag for purification and characterization. In SPR experiment, it was found that the negative control De4, with TrxA fused, did not show binding to any of the ARD protein in TRPV1, TRPV2, or TRPV3 (Figure 3I), indicating TrxA did not affect the binding of the designed protein to ARD. In order to obtain the designed protein with penetrating peptide, the glutathione S‐transferase‐tobacco etch virus‐TAT‐De3 vector was constructed, which was digested and purified to obtain the target protein.

### Calcium Imaging

Transiently transfected HEK293 cells seeded on 25 mm coverslips were washed twice with an extracellular solution (ECS) containing 140 × 10^−3^ m NaCl, 5 × 10^−3^ m KCl, 1 × 10^−3^ m MgCl_2_, 1.8 × 10^−3^ m CaCl_2_, 10 × 10^−3^ m glucose, and 15 × 10^−3^ m hydroxyethylpiperazine ethane sulfonic acid (pH 7.4), followed by incubation in 2 mL of ECS supplemented with 2 × 10^−6^ m Fluo‐4/AM (Kd for Ca^2+^ at 345 × 10^−9^ m) and 0.05% Pluronic F‐127 (both from Molecular Probes) at room temperature for 60 min. Probenecid (2 × 10^−3^ m) was included in all solutions to prevent Fluo‐4 leakage from cells. At the end of incubation, cells were washed three times with ECS and incubated in the same solution for another 20 min at room temperature to complete the intracellular hydrolysis process of the acetoxymethyl (AM) ester, which converts the nonfluorescent Fluo‐4/AM into the fluorescent version Fluo‐4.

Coverslip with dye‐loaded cells was placed in the quick‐release magnetic chamber (Warner) and mounted on the stage of a Nikon Eclipse TE2000‐U microscope system equipped with a Roper Cascade 128B CCD camera. Fluo‐4 was excited by an Argon laser with a filter set of Z488/10 (excitation), z488rdc (dichroic) and recorded through an emission filter HQ500lp (all from Chroma). The duration of light exposure was controlled by a computer‐driven mechanical shutter (Uniblitz). Cell images were acquired sequentially with an exposure period of 200 ms at an interval of 1 s. The shutter and the camera were controlled and synchronized by MetaMorph software (Universal Imaging). Cells pretreated with 1 × 10^−6^ m thapsigargin during the dye‐loading step (aiming to deplete endoplasmic reticulum Ca^2+^ store) did not exhibit noticeable difference in fluorescence intensity or kinetics changes compared to untreated cells.

### Electrophysiology

Patch‐clamp recordings were performed with a HEKA EPC10 amplifier controlled by PatchMaster softerware (HEKA). Whole‐cell recordings at ± 80 mV were used to test whether an mutant channel was functional. Patch pipettes were prepared from borosilicate glass and fire‐polished to resistance of ≈4 MΩ. For whole‐cell recording, serial resistance was compensated by 60%. A solution with 130 × 10^−3^ m NaCl, 10 × 10^−3^ m glucose, 0.2 × 10^−3^ m ethylene diamine tetraacetic acid and 3 × 10^−3^ m Hepes (pH 7.2) was used in both bath and pipette for concentration response curve measurements in TRPV1, TRPV2, and TRPV3. To measure calcium‐dependent ligand‐induced desensitization and tachyphylaxsis, 2 × 10^−3^ m CaCl_2_ was added to the solution. Membrane potential was clamped at ± 80 mV. Current was sampled at 10 kHz and filtered at 2.9 kHz. All recordings were performed at room temperature (22 °C) with the maximum variation of 1 °C. The capsaicin or 2‐aminoethoxydiphenyl borate (APB) concentration‐dependent activation curves were fit to a Hill equation to obtain the EC50 value and the slope factor.

Ligands were perfused to membrane patch by a gravity‐driven system (RSC‐200, Bio‐Logic). Bath and ligand solution were delivered through separate tubes to minimize the mixing of solutions. Patch pipette was placed in front of the perfusion tube outlet.

For recording the heat activation of TRPV1, temperature control was achieved by perfusion of preheated or room temperature bathing solution. Hot bathing solution was maintained at expected temperature with an SH‐27B in‐line solution heater controlled by a TC‐324C temperature controller (Warner). A TA‐29 miniature bead thermistor (Harvard Apparatus) was placed right next to the pipette to ensure accurate monitoring of local temperature.

The current‐temperature relationship was used to determine activation threshold temperature and to characterize thermodynamic properties of heat activation.^[^ ^37^ ^]^ The raising phase of the current‐temperature curve recorded from cells expressing wildtype or mutant TRPV1 channels showed two temperature‐dependent phases, a less temperature‐dependent phase at lower temperatures followed by a higher temperature‐dependent phase at higher temperatures. Each phase was fitted to a linear function. The temperature at the intersection of the two lines was taken as the takeoff temperature and was defined as the activation threshold temperature.

To calculate the change of enthalpic (Δ*H*) and entropic (Δ*S*) due to the temperature‐driven activation of TRPV1, Van't Hoff plots were constructed and they were fitted to the equation ln *K*_eq_ = −Δ*H*/*RT* + Δ*S*/*R* whereby *R* represents the gas constant, *T* represents the temperature in Kelvin, *K*_eq_ represents the equilibrium constant measured from the heat‐driven TRPV1 open probability, *K*_eq_ = *P*_o_/(1 − *P*_o_). The TRPV1 open probability induced by capsaicin at saturated concentration was served as the maximum open probability.

### Animals

Male Sprague–Dawley rats (200–250 g, Charles River Laboratories, Inc.) were housed under a 12 h light–dark cycle and allowed access to food and water ad libitum. The ambient temperature of the holding and testing rooms was ≈22 °C. All procedures involving animals were carried out in strict compliance with the National Institutes of Health and institutional guidelines for the humane care of animals and were approved by the Animal Care and Use Committee of Zhejiang University (Approval ID: ZJU20190100). All efforts were made to minimize both animal numbers and distress within the experiments.

### Animal Behavioral Measurements

A 10 × 10^−3^ m stock solution of MRS1477 was prepared in 100% dimethyl sulfoxide (DMSO) and further diluted in vehicle to 2 µg/100 µL (50 × 10^−6^ m). Capsaicin was prepared as a 100 × 10^−3^ m stock solution in DMSO, stored at −80 °C and was diluted directly into vehicle on the day of the experiments to 4.6 µg/100 µL (150 × 10^−6^ m). Capsaicin‐only injectates contained an equal amount of DMSO as those with MRS1477. All intraplantar injections were made using a 29G × 1/2", 3/10 cc insulin syringe. The experimenter was blinded to the identity of the injectates in the various behavioral experiments.

Thermal hyperalgesia measurement was performed as reported previously.^[^ ^11^, ^12^, ^13^, ^14^, ^15^, ^16^, ^17^, ^18^, ^19^, ^20^, ^21^, ^22^, ^23^, ^24^, ^25^, ^26^, ^27^, ^28^, ^29^, ^30^, ^31^, ^32^, ^33^, ^34^, ^35^, ^36^, ^37^, ^38^ ^]^ Briefly, unrestrained rats were placed on a clear glass platform which a light beam was applied onto the plantar hind paws of the animals under a small plastic cage which allowed them to move freely. The thermal nociceptive response was defined as the latency between light stimulus onset and paw withdrawal using a feedback‐controlled shutdown unit. The intensity of the light stimulus was set such that naive rats responded with a latency of ≈5 s. Each paw was tested one time. In the absence of a response within a predetermined maximum latency (30 s), the test was terminated to prevent tissue damage. On the day of testing, rats were allowed to habituate for at least 30 min prior to thermal stimulation. Rats were tested prior to intradermal injection to establish a baseline, then at 2, 24, 48, 72 h, 7, 16, and 21 d postinjection.

### Statistics

All experiments have been independently repeated for at least three times. All statistical data are given as mean ± standard error of mean. Two‐sided Student's *t*‐test was applied to examine the statistical significance. N.S. indicates no significance. *, ** and ***, *p* < 0.05, *p* < 0.01, and *p* < 0.001, respectively.

## Conflict of Interest

The authors declare no conflict of interest.

## Author Contribution

L.X. and H.Z. contributed equally to this work. F.Y., L.Z.X., Y.H.T., H.Z., Y.F.W., X.C.L., and Z.Y.Z. conducted the experiments including patch‐clamp recording, protein expression, SPR, and animal behavior tests. F.Y. designed the protein modulators. C.M. supervised protein expression and purification. V.Y.‐Y. supervised protein design and revised the manuscript. J.Z. and F.Y. conceived and supervised the project and prepared the paper. J.Z., V.Y.‐Y. S.L.Y., and F.Y. participated in data analysis and paper writing.dfafasdfasdf

## Supporting information

Supporting Information

## Data Availability

All data needed to evaluate the conclusions in the paper are present in the paper and/or the Supporting Information. Additional data available from authors upon request.
