# Supplementary material for: De Novo Design of Peptidic Positive Allosteric Modulators Targeting TRPV1 with Analgesic Effects
Source: Adv Sci (Weinh). 2021 Jul 11;8(17):2101716. doi: 10.1002/advs.202101716 (PMC8425881; doi:10.1002/advs.202101716)
Supplement: Supplementary file 1 — Supporting Information [file ADVS-8-2101716-s001.pdf]

© 2021 Wiley-VCH GmbH

ADVANCED SCIENCE
Open Access

Supporting Information

for *Adv. Sci.*, DOI: 10.1002/advs.202101716

De novo design of peptidic positive allosteric modulators targeting TRPV1 with analgesic effects

Lizhen Xu[1,6,7,8], Heng Zhang[1,6,7,8], Yunfei Wang[3], Xiancui Lu[3], Zhenye Zhao[1], Cheng Ma[4], Shilong Yang[3], Vladimir Yarov-Yarovoy[5], Yuhua Tian[2], Jie Zheng[5]*, Fan Yang[1,5,6,7]**

De novo design of peptidic positive allosteric modulators targeting TRPV1 with analgesic effects

Lizhen Xu[1,6,7,8], Heng Zhang[1,6,7,8], Yunfei Wang[3], Xiancui Lu[3], Zhenye Zhao[1], Cheng Ma[4], Shilong Yang[3], Vladimir Yarov-Yarovoy[5], Yuhua Tian[2]*, Jie Zheng[5]*, Fan Yang[1,5,6,7]*

[1]Kidney Disease Center, First Affiliated Hospital and Department of Biophysics, Zhejiang University School of Medicine, Hangzhou 310058 Zhejiang, China;

[2]Qingdao University School of Pharmacy, Qingdao, Shandong, China;

[3]College of Wildlife and Protected Area, Northeast Forestry University, Harbin 150040, China

[4]Protein facility, School of Medicine, Zhejiang University

[5]Department of Physiology and Membrane Biology, University of California, Davis, School of Medicine, Davis CA 95616, USA;

[6]Alibaba-Zhejiang University Joint Research Center of Future Digital Healthcare

[7]MOE Frontier Science Center for Brain Science & Brain-Machine Integration, Zhejiang University

[8]These authors contributed equally to this work.

*Correspondence should be sent to Yuhua Tian (yhtian05250@qdu.edu.cn), Jie Zheng (jzheng@ucdavis.edu) or Fan Yang (Lead contact, fanyanga@zju.edu.cn)

SUPPLEMENTARY FIGURE LEGENDS

Figure S1. Flowchart illustrating the major steps in the our OHCA stratagem for designing binders to the ARD. The optimization steps in OHCA as compared to the original hotspot centric design method were colored in blue.

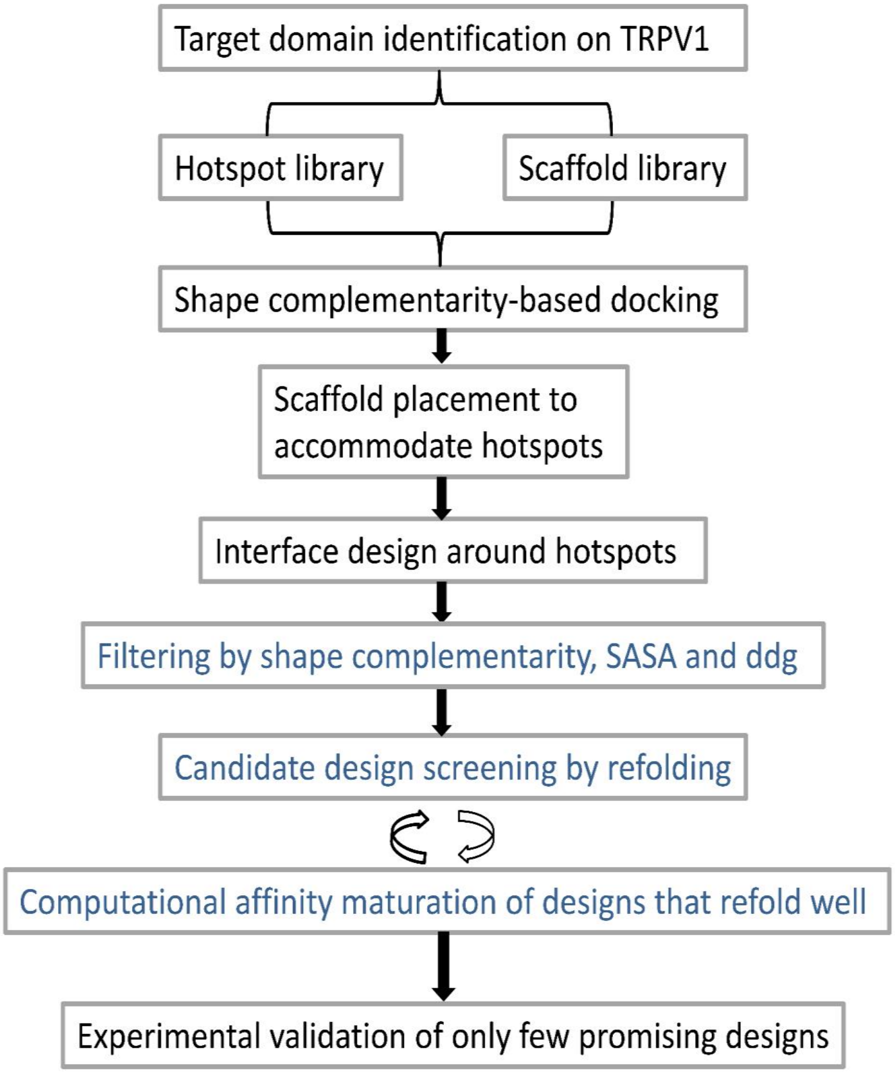

Figure S2. Refolding profiles of the candidate designs. The designed structure was set as the reference to calculate the RMSD of the models generated by *ab initio* refolding.

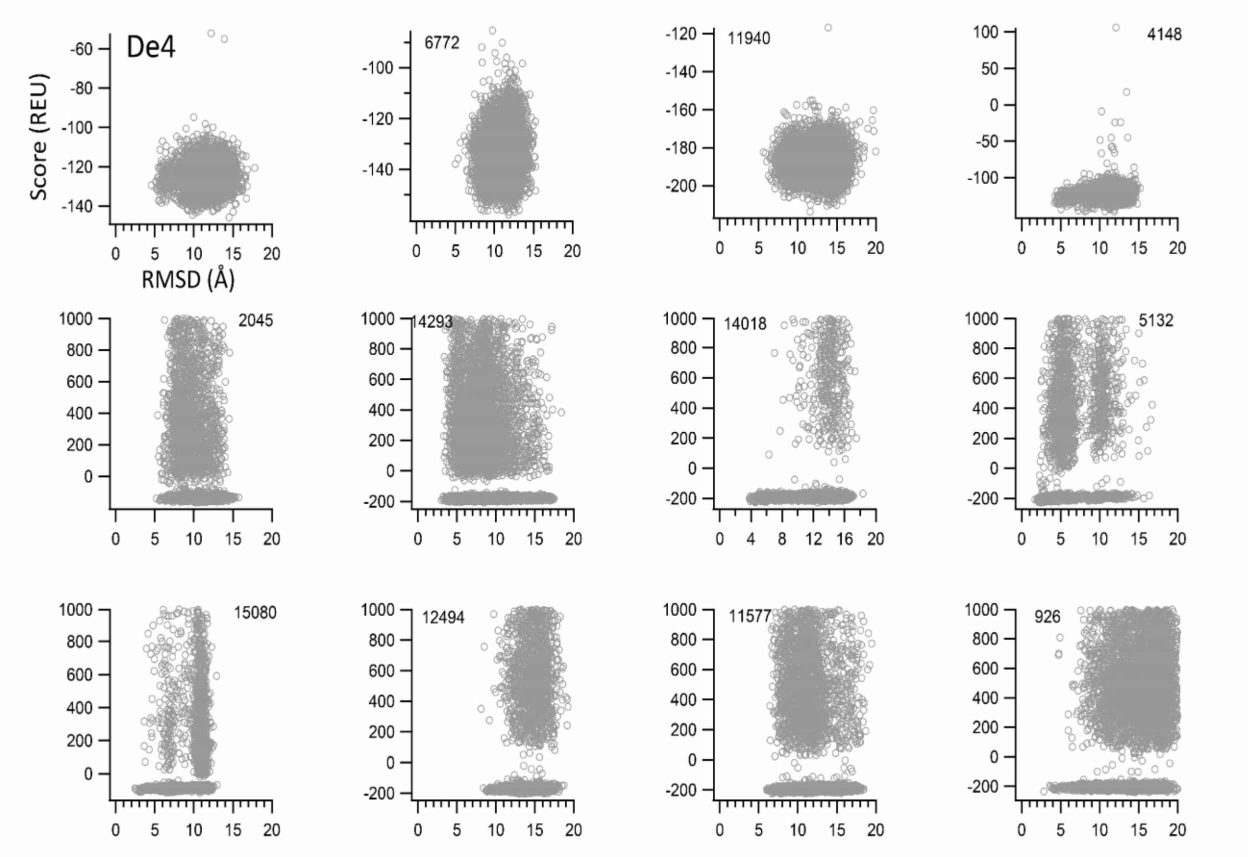

Figure S3. Expression and purification of proteins. (A) The protein supernatant liquid was applied to a 1 mL column of chelating Sepharose to get the flow through (FT). Following the application of the protein sample, the column was extensively washed with different concentrations of imidazole. The protein was resolved by SDS-polyacrylamide gel electrophoresis followed by Coomassie blue staining. The band corresponding to De1 was indicated by black arrows. (B) Purified De1 was resolved by SDS-polyacrylamide gel electrophoresis followed by Coomassie blue staining. (C) The protein supernatant liquid was applied to a 1 mL column of chelating Sepharose to get the flow through (FT). Following the application of the protein sample, the column was extensively washed with different concentrations of imidazole. The protein was resolved by SDS-polyacrylamide gel electrophoresis followed by Coomassie blue staining. The band corresponding to De3 was indicated by black arrows. (D) Purified De3 was resolved by SDS-polyacrylamide gel electrophoresis followed by Coomassie blue staining. (E) The protein supernatant liquid was applied to a 1 mL column of chelating Sepharose to get the flow through (FT). Following the application of the protein sample, the column was extensively washed with different concentrations of imidazole. The protein was resolved by SDS-polyacrylamide gel electrophoresis followed by Coomassie blue staining. The band corresponding to De4 was

indicated by black arrows. (F) Purified De4 was resolved by SDS-polyacrylamide gel electrophoresis followed by Coomassie blue staining. (G) Size exclusion chromatography of De1 on Superpose 6 (GE Healthcare). (H) Size exclusion chromatography of De3 on Superpose 6 (GE Healthcare). (I) Size exclusion chromatography of De4 on Superpose 6 (GE Healthcare). (J) The protein supernatant liquid was applied to a 1 mL column of chelating Sepharose to get the flow through(FT). Following the application of the protein sample, the column was extensively washed with different concentrations of imidazole. The protein was resolved by SDS-polyacrylamide gel electrophoresis followed by Coomassie blue staining. The band corresponding to GST-Tev-TAT-De3 was indicated by black arrows. (K) Purified GST-Tev-TAT-De3 was resolved by SDS-polyacrylamide gel electrophoresis followed by Coomassie blue staining. (L) Lane 1：GST-Tev-TAT-De3 cleaved by TEV protease, 4°, 16h, then resolved by SDS-polyacrylamide gel electrophoresis followed by Coomassie blue staining. Lane 2: Purified TAT-De3 was resolved by SDS-polyacrylamide gel electrophoresis followed by Coomassie blue staining. (M) Purified TRPV1-ARD was resolved by SDS-polyacrylamide gel electrophoresis followed by Coomassie blue staining. (N) Purified TRPV2-ARD was resolved by SDS-polyacrylamide gel electrophoresis followed by Coomassie blue staining. (O) Purified TRPV3-ARD was resolved by SDS-polyacrylamide gel electrophoresis followed by Coomassie blue staining.

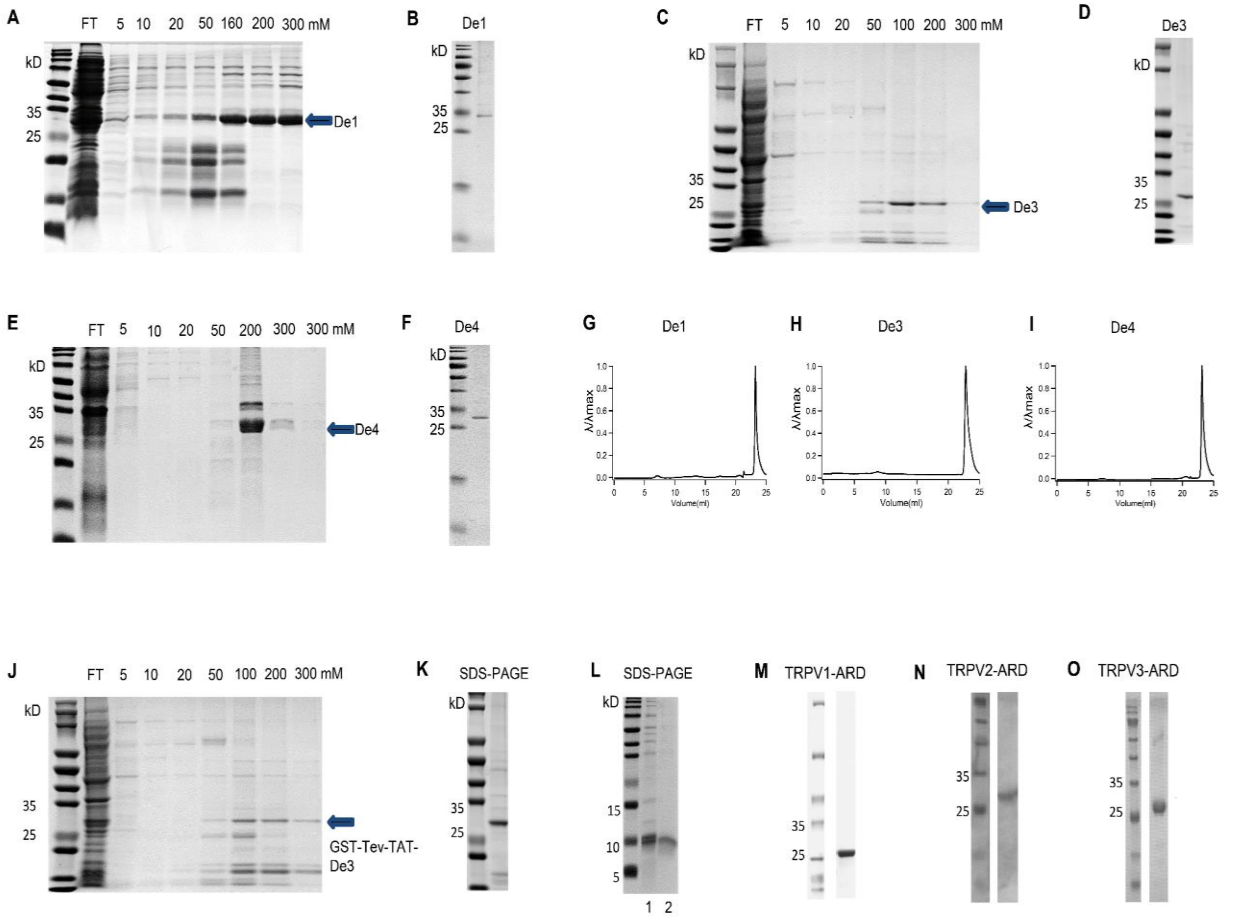

Figure S4. Amino acid sequence alignment of ARDs in TRPV1, TRPV2 and TRPV3. The alignment started from residue 110 in ARD of TRPV1. Individual ankyrin repeat was indicated by arrows in black. Residues important for positive allosteric modulation of TRPV1 by *De1* were indicated by arrows in red.

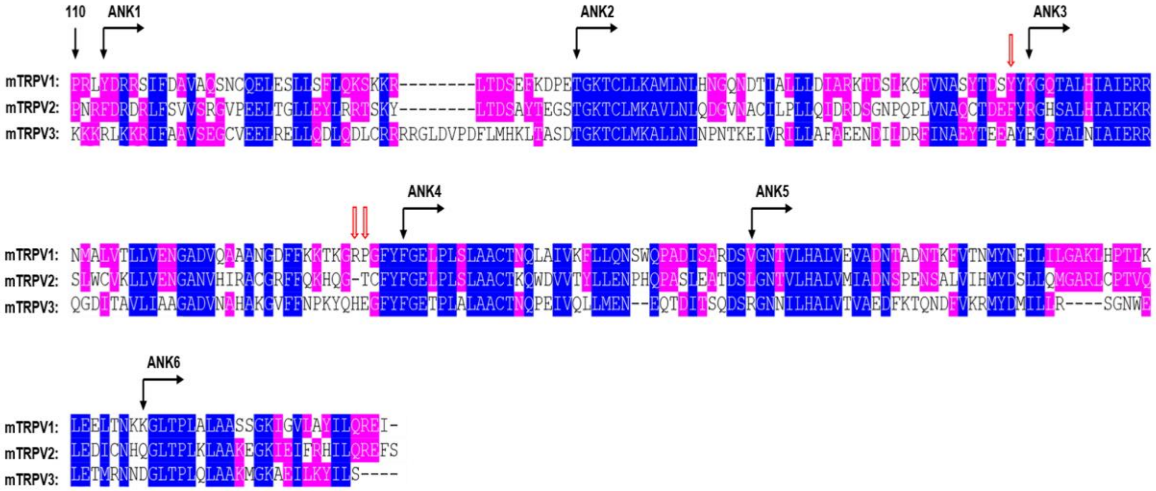

Figure S5. *TAT-De3* binds to the ARD without disrupting cell membrane. (A) SPR signal traces of *TAT-De3* at distinct concentration levels bound to the ARD fixed on the sensor chip. (B) Fluorescence and bright field imaging of cells expressing TRPV1-YFP (pseudo colored in green) before and 30 min after adding 300 µM *TAT-De3* to the bath solution. No apparent disruption of cell membrane was observed.

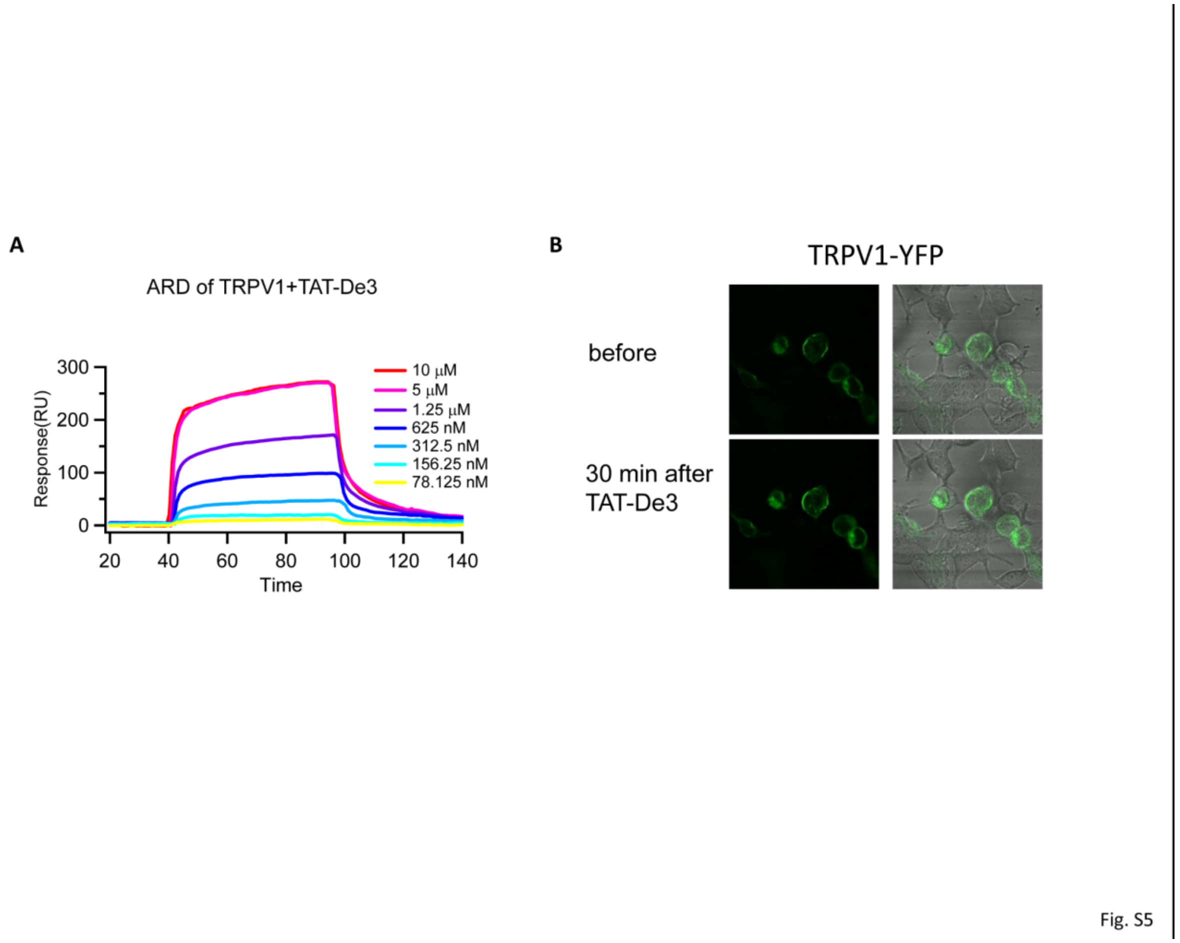

Supplementary Table 1. Information of the designed binder proteins to the ARD of TRPV1.

Design name	Scaffold PDB	Number of residues	Number of mutations	Total score (REU)	ddg (REU)	Total SASA (Å)	Hydrophobic area (Å)	Shape complementarity
De1	1QZM	93	24	-497.304	-31.13	1930.48	1367.27	0.6743
De2	1QZM	93	26	-567.079	-40.64	2253.36	1689.67	0.6853
De3	3ONJ	96	26	-608.476	-45.18	2991.22	2049.31	0.6355
De4	2HDZ	65	22	-408.786	-37.35	2311.28	1843.63	0.7126

Supplementary Table 2. Apparent K_D and FRET $Ratio_{max}$ measured from FRET imaging of the design-TRPV1 interactions in cells. n.a., not available.

Designs	Apparent K_D	FRET Ratio max
De1	$1.2*10^{5}$	3.86
De2	$7.5*10^{4}$	2.35
De3	$1.1*10^{4}$	1.99
De4	$9.5*10^{9}$	n.a.

Supplementary Table 3A. The primary amino acid sequences of the designed proteins. (A) The primary amino acid sequences of the designed binder proteins and the ARD of TRPV1, TRPV2 and TRPV3 channels. The various modules of candidate designed binder proteins were highlighted in different colors. (B) TrxA, 6xHis, S-Tag, thrombin site, enterokinase site, TEV site and TAT sequence were colored in red, purple, orange, green, light green, dark blue and grey, respectively. Candidate designed binder proteins, TRPV1-ARD and the linkers were colored in blue, light blue and black, respectively. ARD protein was first cleaved by TEV protease, and then purified through Ni-chelating affinity chromatography.

Protein	Primary sequence
**De1**	MSDKIIHLTDDSFDTDVLKADGAILVDFWAEWCGPCKMIAPILDEIADEYQGKLTVAKLNIDQNPGTAPKYGIRGIPTLLLFKNGEVAATKVGALSKGQLKEFLDANLAGSGSGHMHHHHHHSSGLVPRGSGMKETAAAKFERQHMDSPDLGTDDDDKAMADIGSNGYSSEDKLRIAKRHLLPKQIERNALKKGELFVSEFAILGIILFYTDEAGVRGLEREISKLCRKAVKQLLLDKSTTSSHASGFHLHDYLGVMR
**De2**	MSDKIIHLTDDSFDTDVLKADGAILVDFWAEWCGPCKMIAPILDEIADEYQGKLTVAKLNIDQNPGTAPKYGIRGIPTLLLFKNGEVAATKVGALSKGQLKEFLDANLAGSGSGHMHHHHHHSSGLVPRGSGMKETAAAKFERQHMDSPDLGTDDDDKAMADIGSNGYWSEDKERIAKRHLLPKQIERNALKKGELKVWEFAIFGIILFYTWEAGVRGLEREISKLCRKAVKQLLLDKSTTRSWASGFHLHDYLGVMR
**De3**	MSDKIIHLTDDSFDTDVLKADGAILVDFWAEWCGPCKMIAPILDEIADEYQGKLTVAKLNIDQNPGTAPKYGIRGIPTLLLFKNGEVAATKVGALSKGQLKEFLDANLAGSGSGHMHHHHHHSSGLVPRGSGMKETAAAKFERQHMDSPDLGTDDDDKAMADIGSSLLDSYKLDFWKTSTNAENSLHEAPSQPLSQRNTTLKHVEQQQDELFDLLDQMDVEVNNSIGRDADRRWYLFELWFWKISIEEEIKRPLQSLVDSG
**De4**	MSDKIIHLTDDSFDTDVLKADGAILVDFWAEWCGPCKMIAPILDEIADEYQGKLTVAKLNIDQNPGTAPKYGIRGIPTLLLFKNGEVAATKVGALSKGQLKEFLDANLAGSGSGHMHHHHHHSSGLVPRGSGMKETAAAKFERQHMDSPDLGTDDDDKAMADIGSLPESPRFAQKIWQQSVIGDYLARFKNDRVKALKAMEDSSNNSSMHRFAEFFAKAFEDLHRYLEEL
**TAT-De3**	YGRKKRRQRRRSLLDSYKLDFWKTSTNAENSLHEAPSQPLSQRNTTLKHVEQQQDELFDLLDQMDVEVNNSIGRDADRRWYLFELWFWKISIEEEIKRPLQSLVDSG
**TRPV1-ARD**	HHHHHHSSGENLYFQGLYDRRSIFDAVAQSNCQELESLLPFLQRSKKRLTDSEFKDPETGKTCLLKAMLNLHNGQNDTIALLLDVARKTDSLKQFVNASYTDSYYKGQTALHIAIERRNMTLVTLLVENGADVQAAANGDFFKKTKGRPGFYFGELPLSLAACTNQLAIVKFLLQNSWQPADISARDSVGNTVLHALVEVADNTVDNTKFVTSMYNEILILGAKLHPTLKLEEITNRKGLTPLALAASSGKIGVLAYILQREIH
**TRPV2-ARD**	HHHHHHSSGENLYFQGRFDRDRLFSVVSRGVPEELTGLLEYLRRTSKYLTDSAYTEGSTGKTCLMKAVLNLQDGVNACILPLLQIDRDSGNPQPLVNAQCTDEFYRGHSALHIAIEKRSLWCVKLLVENGANVHIRACGRFFQKHQGTCFYFGELPLSLAACTKQWDVVTYLLENPHQPASLEATDSLGNTVLHALVMIADNSPENSALVIHMYDSLLQMGARLCPTVQLEDICNHQGLTPLKLAAKEGKIEIFRHILQREFS
**TRPV3-ARD**	HHHHHHSSGENLYFQGQKKKRLKKRIFAAVSEGCVEELRELLQDLQDLCRRRRGLDVPDFLMHKLTASDTGKTCLMKALLNINPNTKEIVRILLAFAEENDILDRFINAEYTEEAYEGQTALNIAIERRQGDITAVLIAAGADVNAHAKGVFFNPKYQHEGFYFGETPLALAACTNQPEIVQLLMENEQTDITSQDSRGNNILHALVTVAEDFKTQNDFVKRMYDMILLRSGNWELETMRNNDGLTPLQLAAKMGKAEILKYILS

Supplementary Table 3B.

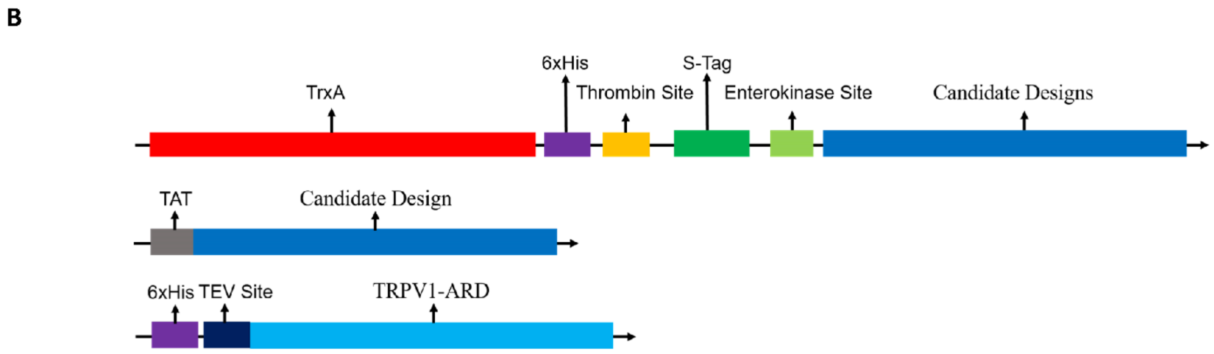

SUPPLEMENTARY SCRIPTS

Supplementary Script 1. Relax of the ADR structure.

```
#!/bin/bash
#$ -S /bin/bash
#$ -e /home/fanyang/work/constrained_relax_2PNN-2PNN-_/$TASK_ID/
#$ -o /home/fanyang/work/constrained_relax_2PNN-2PNN-_/$TASK_ID/

/home/fanyang/rosetta_source/bin/relax.linuxgccrelease \
-in:path:database /home/fanyang/rosetta_database \
-in:file:s /home/fanyang/projects/input_files/2PNN/2PNN_cleaned_fy.pdb \
-ignore_unrecognized_res \
-relax:fast \
-relax:constrain_relax_to_start_coords \
-relax:coord_constrain_sidechains \
-relax:ramp_constraints false \
-ex1 \
-ex2 \
-use_input_sc \
-correct \
-nstruct 100 \
-out:prefix constrained-relax- \
-out:file:silent /home/fanyang/work/constrained_relax_2PNN-2PNN-
_/${SGE_TASK_ID}/constrained-relax-_2PNN_${SGE_TASK_ID}.silent \
-out:file:silent_struct_type binary \
-mute all
```

Supplementary Script 2. Building the inverse rotamer library.

```
<dock_design>
	<SCOREFXNS>
		<stub_dock weights=talaris2014 />
	</SCOREFXNS>
	<FILTERS>
		<EnergyPerResidue name=energy scorefxn=stub_dock pdb_num=1B
energy_cutoff=1/>
		<Ddg name=ddg scorefxn=stub_dock threshold=-1 repack=0/>
	</FILTERS>
	<MOVERS>
		<TryRotamers name=try pdb_num=1B /> list residues the backbones of which
are to be ignored in energy evaluations under the shove flag
		RepackMinimize name=rpk repack_partner1=1 repack_partner2=0
design_partner1=0 design_partner2=0 minimize_bb=0 minimize_rb=0 minimize_sc=1
	</MOVERS>
	<APPLY_TO_POSE>
	</APPLY_TO_POSE>
	<PROTOCOLS>
		<Add mover_name=try/>
		add mover_name=rpk
		<Add filter_name=energy/>
		<Add filter_name=ddg/>
	</PROTOCOLS>
</dock_design>
```

Supplementary Script 3. Clean of the protein structures selected from PDB database.

```
#!/bin/bash
FOLDER_A=/media/Data_Ubuntu/PatchDock/scaffold_0402
FOLDER_B=/media/Data_Ubuntu/PatchDock/scaffold_cleaned_0402

for file_a in ${FOLDER_A}/*; do
	file_a_prefix=${file_a%.*}
	file_prefix=${file_a_prefix#${FOLDER_A}/*}
	echo $file_prefix
	sudo cleanPdb.pl -pdbfile ${FOLDER_A}/$file_prefix.pdb >
${FOLDER_B}/$file_prefix.pdb
done
```

Supplementary Script 4. Prepack of the cleaned protein structures selected from PDB database.

```
#!/bin/bash
FOLDER_A=/media/Data_Ubuntu/PatchDock/scaffold_0405_cleaned

for file_a in ${FOLDER_A}/*; do
	file_a_prefix=${file_a%.*}
	file_prefix=${file_a_prefix#${FOLDER_A}/*}
	echo $file_prefix
	/home/fan/rosetta_source/bin/rosetta_scripts.linuxgccrelease -ex1 -ex2aro -
database /home/fan/rosetta_source/rosetta_database -ignore_unrecognized_res -
in:file:fullatom -in:file:s /home/fan/PatchDock/PDB/scaffold_PDB_1/$file_prefix.pdb -
parser:protocol /home/fan/rosetta_source/input_files/prepack_2PNN/ppk.xml -overwrite
-out:output
	sudo mv $file_prefix*.pdb $file_prefix.pdb
	sudo cp $file_prefix.pdb
/media/Data_Ubuntu/PatchDock/scaffold_0405_cleaned_prepacked/
	rm -r $file_prefix.pdb
done
```

Supplementary Script 5. Fusion of scaffold proteins with the hotspots.

```
#!/bin/bash
#$ -S /bin/bash
#$ -e /home/fanyang/work/Placestub_PheHS2_PheHS1_2PNN_0511-2PNN-_/$TASK_ID/
#$ -o /home/fanyang/work/Placestub_PheHS2_PheHS1_2PNN_0511-2PNN-_/$TASK_ID/

n=(${SGE_TASK_ID}-1)*100+1
for ((i=1; i<101; i=i+1))
do /home/fanyang/rosetta_source/bin/rosetta_scripts.static.linuxgccrelease \
-in:path:database /home/fanyang/rosetta_database \
-in:file:fullatom \
-ex1 \
-ex2 \
-in:file:s /home/fanyang/projects/input_files/2PNN/Docked_0510/$[${i}+${n}].pdb \
-out:file:scorefile mscore_$[${i}+${n}].fsc \
-nstruct 50 \
-out:file:silent /home/fanyang/work/Placestub_PheHS2_PheHS1_2PNN_0511-2PNN-
_/${SGE_TASK_ID}/twoHS_placestubs_2PNN_${SGE_TASK_ID}.silent \
-out:file:silent_struct_type binary \
-mute all \
-parser:protocol
/home/fanyang/projects/input_files/2PNN/TwoResidueHotspot_fy_0511.xml
done
```

TwoResidueHotspot_fy_0511.xml:

```
<ROSETTASCRIPTS>
      <TASKOPERATIONS>
            <PreventRepacking name=prevent_repacking_Y84 resnum=84/>
            <PreventRepacking name=prevent_repacking_Y89 resnum=89/>
      </TASKOPERATIONS>
      <SCOREFXNS>
            <stub_docking_low weights=interchain_cen hs_hash=10.0/>
            <ddg_scorefxn weights=standard patch=score12 hs_hash=0.0/>
            <score12_coordcst weights=standard patch=score12 hs_hash=0.0>
                  <Reweight scoretype=coordinate_constraint weight=1.0/>
            </score12_coordcst>
  </SCOREFXNS>
   <FILTERS>
             <Ddg name=ddg threshold=-10 scorefxn=ddg_scorefxn repeats=3/>
             <Sasa name=sasa threshold=1200/>
             <CompoundStatement name=ddg_sasa> for the loop over filter
                        <AND filter_name=ddg/>
                        <AND filter_name=sasa/>
                  </CompoundStatement>
                  <TerminusDistance name=termini distance=3/>
       <AtomicContact name=touch_125 residue1=125A distance=5/>
       <AtomicContact name=touch_89 residue1=89A distance=5/>
       <AtomicContact name=touch_84 residue1=84A distance=5/>
       <AtomicContact name=touch_53 residue1=53A distance=5/>
       <AtomicContact name=touch_97 residue1=97A distance=5/>
                  <CompoundStatement name=touch_Trp>
                        <AND filter_name=touch_125/>
                        <AND filter_name=touch_89/>
                             <AND filter_name=touch_97/>
                  </CompoundStatement>
                      <CompoundStatement name=touch_Leu>
                             <AND filter_name=touch_84/>
                             <AND filter_name=touch_53/>
                      </CompoundStatement>
   </FILTERS>
   <MOVERS>
      <Docking name=dock fullatom=0 local_refine=0 score_low=stub_docking_low/>
                  <RepackMinimize name=des1 minimize_bb=0 minimize_rb=1
scorefxn_repack=soft_rep scorefxn_minimize=score_docking
interface_cutoff_distance=10.0/>
                  <RepackMinimize name=des2 minimize_bb=0 minimize_rb=1
interface_cutoff_distance=10.0/>
                  <RepackMinimize name=des3 minimize_bb=1 minimize_rb=1
interface_cutoff_distance=10.0/>
```

```
                    <SaveAndRetrieveSidechains name=srsc/>
                    <BackrubDD name=br partner1=0 partner2=1
interface_distance_cutoff=10.0/>
                    <PlaceStub name=place_top minimize_rb=1 triage_positions=1
stubfile="/home/fanyang/projects/input_files/2PNN/HS_lib/PheHS2_IR.pdb"
stub_energy_threshold=10 final_filter=ddg_sasa hurry=1 max_cb_dist=4.0
task_operations=prevent_repacking_Y84>
                              <StubMinimize>
                                    <Add mover_name=br/>
                              </StubMinimize>
                              <DesignMovers>
                                    <Add mover_name=srsc/>
                                    <Add mover_name=des1 coord_cst_std=1.0/>
                                    <Add mover_name=des2 coord_cst_std=1.5/>
                                    <Add mover_name=br/>
                                    <Add mover_name=des3/>
                              </DesignMovers>
                    </PlaceStub>
                    <PlaceStub name=place_trp place_scaffold=1 triage_positions=0
stubfile="/home/fanyang/projects/input_files/2PNN/HS_lib/PheHS1_IR.pdb"
task_operations=prevent_repacking_Y89 add_constraints=1 minimize_rb=1 hurry=1
score_threshold=10.0 stub_energy_threshold=3.0 max_cb_dist=4.0 leave_coord_csts=1
task_operations=prevent_repacking_Y84>
                              <StubMinimize>
                                    <Add mover_name=br/>
                              </StubMinimize>
                              <DesignMovers>
                                    <Add mover_name=place_top coord_cst_std=1.0/>
                              </DesignMovers>
                              </PlaceStub>
             </MOVERS>
      <APPLY_TO_POSE>
             <SetupHotspotConstraints
stubfile="/home/fanyang/projects/input_files/2PNN/HS_lib/PheHS2_PheHS1_IR.pdb"
cb_force=0.5/>
      </APPLY_TO_POSE>
      <PROTOCOLS>
                    <Add mover_name=dock filter_name=termini/>
                          <Add mover_name=place_trp/>
                          <Add filter_name=ddg/>
                          <Add filter_name=sasa/>
      </PROTOCOLS>
</ROSETTASCRIPTS>
```

Supplementary Script 6. In silico affinity maturation of candidate designes.

```
/home/fanyang/rosetta_source/bin/rosetta_scripts.static.linuxgccrelease

-database /home/fan/rosetta_source/rosetta_database
-ignore_unrecognized_res
-in:file:fullatom
-ex1
-ex2
-in:file:s
/media/Data2/Academic/Rosetta/Rosetta_Papers/ProteinDesign/supplement_Science2011_Bak
er/AllHADesigns/HB36.pdb
-parser:protocol
/media/Data2/Academic/Rosetta/Rosetta_script/xml_scripts_Fan/MulticriterionOptimizati
on_fy.xml
-nstruct 1000
-overwrite
```

MulticriterionOptimization_fy.xml:

```
<dock_design>
        <TASKOPERATIONS>
                <InitializeFromCommandline name=init/>
                <ProteinInterfaceDesign name=pido interface_distance_cutoff=10/>
                <RestrictResidueToRepacking name=restrict256 resnum=256/>
                <RestrictResidueToRepacking name=restrict260 resnum=260/>
                <RestrictAbsentCanonicalAAS name=nohis
keep_aas="ACDEFGIKLMNPQRSTVWY"/>
        </TASKOPERATIONS>
        <SCOREFXNS>
        </SCOREFXNS>
        <FILTERS>
                <Sasa name=hydrophobic_sasa hydrophobic=1 confidence=0/>
                <Sasa name=sasa confidence=0/>
                <Ddg name=ddg confidence=0 repeats=3/>
                <ScoreType name=total_score score_type=total_score threshold=0/>
        </FILTERS>
        <MOVERS>
                <AtomTree name=docking_tree docking_ft=1/>
                <RandomMutation name=random_mutation
task_operations=init,pido,restrict256,restrict260,nohis/>
                <MinMover name=min bb=1 chi=1 jump=1>
                        <MoveMap>
                                <Chain number=1 chi=1 bb=0/>
                        </MoveMap>
                </MinMover>
```

```
		<ParsedProtocol name=agg_mover>
			<Add mover=random_mutation/>
			<Add mover=min/>
		</ParsedProtocol>
		<GenericMonteCarlo name=genericMC mover_name=agg_mover 
filter_name=ddg temperature=0.1 trials=1000>
			<Filters>
				<AND filter_name=total_score temperature=1/>
			</Filters>
		</GenericMonteCarlo>
	</MOVERS>
	<APPLY_TO_POSE>
	</APPLY_TO_POSE>
	<PROTOCOLS>
		<Add mover=docking_tree/>
		<Add mover=genericMC/>
		<Add filter=ddg/>
		<Add filter=sasa/>
	</PROTOCOLS>
</dock_design>
```
